# Leveraging Datathons to Teach AI in Undergraduate Medical Education: Case Study

**DOI:** 10.2196/63602

**Published:** 2025-04-16

**Authors:** Michael Steven Yao, Lawrence Huang, Emily Leventhal, Clara Sun, Steve J Stephen, Lathan Liou

**Affiliations:** 1 Perelman School of Medicine, University of Pennsylvania, Philadelphia, PA, United States; 2 Department of Bioengineering, University of Pennsylvania, Philadelphia, PA, United States; 3 MDplus, New York, NY, United States; 4 Warren Alpert Medical School, Brown University, Providence, RI, United States; 5 Icahn School of Medicine at Mount Sinai, New York, NY, United States; 6 School of Medicine, Case Western Reserve University, Cleveland, OH, United States; 7 School of Medicine and Dentistry, University of Rochester, Rochester, NY, United States; 8 Simon Business School, University of Rochester, Rochester, NY, United States

**Keywords:** data science education, datathon, machine learning, artificial intelligence, undergraduate medical education

## Abstract

**Background:**

As artificial intelligence and machine learning become increasingly influential in clinical practice, it is critical for future physicians to understand how such novel technologies will impact the delivery of patient care.

**Objective:**

We describe 2 trainee-led, multi-institutional datathons as an effective means of teaching key data science and machine learning skills to medical trainees. We offer key insights on the practical implementation of such datathons and analyze experiences gained and lessons learned for future datathon initiatives.

**Methods:**

We detail 2 recent datathons organized by MDplus, a national trainee-led nonprofit organization. To assess the efficacy of the datathon as an educational experience, an opt-in postdatathon survey was sent to all registered participants. Survey responses were deidentified and anonymized before downstream analysis to assess the quality of datathon experiences and areas for future work.

**Results:**

Our digital datathons between 2023 and 2024 were attended by approximately 200 medical trainees across the United States. A diverse array of medical specialty interests was represented among participants, with 43% (21/49) of survey participants expressing an interest in internal medicine, 35% (17/49) in surgery, and 22% (11/49) in radiology. Participant skills in leveraging Python for analyzing medical datasets improved after the datathon, and survey respondents enjoyed participating in the datathon.

**Conclusions:**

The datathon proved to be an effective and cost-effective means of providing medical trainees the opportunity to collaborate on data-driven projects in health care. Participants agreed that datathons improved their ability to generate clinically meaningful insights from data. Our results suggest that datathons can serve as valuable and effective educational experiences for medical trainees to become better skilled in leveraging data science and artificial intelligence for patient care.

## Introduction

The exploration of machine learning (ML), artificial intelligence (AI), and other data science-driven technologies is becoming increasingly popular within clinical medicine [1-5]. Given the rapidly growing presence of ML in health care innovation, it is important for both current and future physicians to understand the fundamentals of ML technology and how they may help inform clinical decision-making.

However, data science and AI education in current medical school curricula are lacking. Despite recent efforts to integrate AI learning objectives into medical education [6-10], few US medical schools have formally integrated AI-based topics into their curricula. Pupic et al [11] and Civaner et al [12] report studies of small self-selected groups of medical students and residents participating in both student- and faculty-led electives covering the fundamental theory behind AI applications for medicine. However, opportunities facilitating real-world experience remain limited [13,14].

One potential method for hands-on AI education popular across many fields of science and engineering is the “datathon,” which is a short competition where teams of students work together to create new solutions to domain-specific challenges through leveraging real-world data and algorithms. Following Daneshvar et al [15], we also make the important distinction between datathons and hackathons. Traditionally, hackathons are product-orientated initiatives where team projects are primarily focused on programming novel products and applications. By contrast, the primary learning objectives for our datathons were to (1) teach student participants how to analyze complex datasets to support clinical insights, and (2) leverage ML models to derive these clinical insights from data. Oyetade et al [16] offer a scoping review of datathons and found that such events help students learn both technical and soft skills and argue that datathon-based pedagogies be incorporated in classroom environments. Silver et al [17] describe a hackathon event for current attendings in clinical practice and found that study participants were better equipped to accelerate specialty-focused innovation after the hackathon. However, similar events specifically designed for medical students and other undergraduate trainees are not well described in the literature.

In this work, we hypothesize that datathons can be an effective training initiative to teach skills in AI to medical trainee participants. To evaluate this hypothesis, we describe 2 datathons hosted by MDplus, a 501(c)3 national student-run nonprofit whose mission is to support and empower future physician-innovators. We describe the structure of the events, present data on educational outcomes, and offer resources and recommendations for putting together similar events in the future. Our results suggest that datathons and similar events may be an effective means for AI education for medical students.

## Methods

### Overview

**Figure 1.**
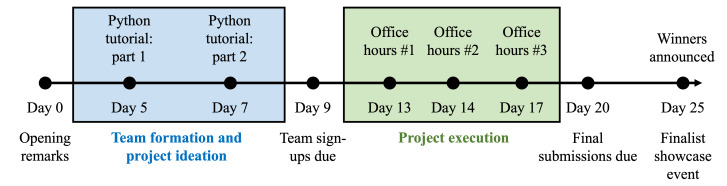
Overview of the datathon event. The MDplus datathon ran for approximately 4 weeks and was loosely divided into two parts: (1) Team formation and project ideation and (2) project execution.

In this section, we detail the logistics of organizing and executing 2 trainee-led datathon events. A number of features distinguish our events from prior work. First, the datathons are trainee-led; all members of the organizing committee were undergraduate medical trainees at the time of the event. Second, the datathons were held digitally over the course of approximately 3 weeks (Figure 1). Finally, the target participant audience of our datathons included current undergraduate medical trainees at institutions granting doctoral degrees. These features of our datathons substantially differentiate them from prior work [15-17], and also affected our design and organization of the events that we detail below.

### Timeline and Participant Recruitment

We, the datathon organizing team, detail 2 datathon events organized by MDplus between 2023 and 2024, herein referred to as “the datathons.” Each datathon ran for approximately 3 weeks (Figure 1), and was organized by the medical trainee-led executive team of MDplus, consisting of a core datathon planning team of 8 medical trainees. To accommodate the participation of medical trainees from across the United States, the entirety of both datathons was held digitally. The MDplus’ Slack community, monthly newsletter, and social media pages (LinkedIn, Instagram, and Twitter) were used to advertise the datathon. Over the span of 3 months prior to the start of the datathon, 2 organizing team members were tasked with recruiting sponsors, mentors, knowledge experts, and judges through the MDplus and personal networks, while 3 organizing team members—all with prior experience working as software engineers prior to medical school—crafted and iterated the educational material and dataset curation for the event. One team member helped publicize the event on social media. Registration for the event was limited to current trainees (ie, medical students, residents, and graduate students) in the United States. To provide a fair learning environment for trainees, our organizing team opted to exclude attending physicians, industry professionals, and individuals with extensive technical backgrounds in software engineering from participation. Participants were asked to form their own teams of 3-5 individuals.

### Datathon Theme and Dataset

Each of the datathons focused on a specific theme to help participants contextualize their projects within a specific application relevant to health care. The theme of the 2024 datathon was responsible generative AI and that of the 2023 datathon was value-based care (VBC). Generative AI is an area of ML that uses technologies such as large language models (LLMs) to create new content by learning patterns from existing human-generated examples [18-20]. While such technologies have the potential to improve health care delivery, recent work has highlighted a growing need to better evaluate how clinicians can use these tools responsibly before real-world integration is possible [21-23]. Separately, VBC refers to a health care delivery model in which providers are held accountable for improving patient outcomes. In a VBC system, providers are often rewarded with incentivized payments based on quality of care, provider performance, and the patient experience [24].

To enable participants to explore projects related to each of these themes, a medical dataset was made available for participants to use in each of our datathons. All datasets were made available via Hugging Face (Hugging Face, Inc), a public repository to facilitate the sharing of ML data and models. In our 2023 VBC datathon, participants were required to use the Medical Information Mart for Intensive Care (MIMIC)-IV dataset [25], a single-site dataset of patient records and admission details. Briefly, the MIMIC-IV dataset contains anonymized patient data aggregated from over 500,000 patients at the Beth Israel Deaconess Medical Center between 2008 and 2019. Variables from this rich dataset include electrocardiograms, medical imaging studies, health records, and patient laboratory values and outcomes, among others. We chose to use this dataset specifically for the datathon because of the following factors:

#### Public Availability

In similar prior events organized by the authors, we found that procuring a real-world dataset of health care data can often be prohibitively expensive or constraining, especially for trainee-led initiatives with limited budgets. To circumvent this problem, we used the MIMIC-IV dataset, which is made publicly available by Johnson et al [25].

#### Real Patient Data and Outcomes

The primary learning objective of our datathons is to teach participants how to derive data-driven insights to affect and ultimately improve patient care. We therefore sought to provide real patient data for participants to explore and use for their projects in alignment with this goal.

#### Prevalence of Prior Work

The vast majority of our participants have minimal (if any) prior experience with programming and data analysis techniques. For this reason, the abundance of prior literature and publicly available coding resources for interacting with the MIMIC-IV dataset helped lower the barrier to participating in the datathon.

#### Multiple Modalities of Data

Many participants have individual academic and personal interests in medicine, and we sought to encourage participants to craft and work on projects that were interesting to them. The abundance of textual, image, biomedical signal, and laboratory data available in the MIMIC-IV dataset was important to make this possible.

All participants in the datathon were required to sign a data use agreement and complete responsible data handling training in order to gain access to the MIMIC-IV dataset. Participating teams were tasked with thinking critically about quantitative methods, conducting appropriate analyses (eg visualization, statistics, and other computational tools), and contextualizing clinical insights into actionable proposals that solve a problem related to VBC for relevant stakeholders.

While organizing for the 2024 generative AI datathon, we found that one limitation of the MIMIC-IV dataset was its size and complexity, making it unwieldy for some participants to work with for their projects. To overcome this challenge while simultaneously retaining the desirable features listed above, our 2024 datathon introduced the concept of datathon “tracks”: teams were able to choose to participate in 1 of 3 tracks within the broader theme of responsible generative AI. Each track was associated with its own dataset: (1) Clinical Documentation track participants used the MTS-Dialog dataset of patient-physician conversation transcripts from Abacha et al [26]; (2) Medical Education track participants used the MedQA dataset of practice medical board examination questions from Jin et al [27]; and (3) Mental Health track participants used the SuicideWatch and Mental Health Collection dataset of tagged social media posts from Ji et al [28]. Participants were allowed to participate in at most 1 of the 3 tracks.

### Resources and Support

An official datathon page [29] was created for participants as a central hub with instructions, registration, and materials for the event. Links to the datathon’s Github Repository were provided with written tutorials and example code, including (1) downloading and overview of the datasets; (2) introduction to Python (Python Software Foundation; offered in both the 2023 and 2024 datathons; see Multimedia Appendix 1); and (3) an introduction to R (R Foundation; offered only in the 2023 datathon). Optional workshops and private Zoom (Zoom Communications, Inc) events with experienced data scientists were offered to participating trainees, including Python and R bootcamps, oral presentation workshops, and a prerecorded Zoom talk with physician experts. The scope of the projects was largely left up to the discretion of individual team members; participant teams were encouraged to leverage the optional workshop sessions and public discussion channels on Slack if they would benefit from discussing potential project ideas with others, although no explicit guidance on project ideation or constraints was given other than all teams had to (1) use the official datathon dataset and (2) work on a project under the broad datathon theme (ie, VBC in 2023 and responsible generative AI in 2024) and track. No tutorials or structured datathon programming were provided for teaching participants how to use GitHub, GitLab, Microsoft Excel, or other computing tools. Communication and announcements throughout the datathon were conducted through Slack.

### Submission Requirements and Judging Criteria

In the 2023 VBC datathon, teams were asked to submit a written technical report of their work without restrictions on the word count and were asked to record a 5-minute-long oral presentation highlighting key contributions and findings. Participants were free to use any programming language or software to perform their analysis. In the 2024 generative AI datathon, teams were asked to submit a 1-page extended abstract with at most 1 figure and unlimited references and a written technical report without word count restrictions. Judging criteria in both datathons included statistical rigor, relevance to the datathon theme (VBC), creativity of visualization and analysis, and team diversity (Multimedia Appendix 2).

### Final Showcase Event

In the 2023 VBC datathon, an internal set of 4 blinded judges composed of members of the MDplus datathon organizing committee evaluated the initial anonymized submissions and selected 7 finalist teams to present at the finalist datathon showcase event. Each team played their recorded 5-minute oral presentations and were allotted 2 minutes immediately after for responding to judge questions. A panel of 5 judges—recruited for their diverse range of expertise in the VBC space—evaluated the finalists’ submissions. In total, 3 of the judges are health care executives, 4 are practicing clinicians, and 1 is a product manager.

In the 2024 generative AI datathon, an internal set of 3 blinded judges composed of members of the organizing committee evaluated the 14 initial anonymized team submissions and selected 8 finalist teams to present at the final datathon showcase. Finalist teams were invited to a 2-hour finalist showcase event where they were each allotted 8 minutes for a live oral presentation followed by 2 minutes of question answering with the judges. We recruited a panel of 4 judges to evaluate the finalist submissions: 1 judge is a software engineer at a health care company, 1 judge is a postdoctoral fellow in a health care AI lab, and 2 judges are practicing physicians in the United States. In general, we found the live oral presentations to be better received by the judges and audience members than playing prerecorded presentations.

### Postdatathon Survey

Upon the conclusion of each datathon, an anonymous 16-question open survey (Multimedia Appendix 3) was electronically sent to all registered participants that submitted a final project via both Slack and email; this survey study was exempted by the University of Pennsylvania Institutional Review Board (protocol #856530). The survey was created in close collaboration with an attending physician at a US academic medical institution with expertise in medical education and assessing educational outcomes and was piloted within the datathon organizing team prior to the public release of the survey. Participants were requested to complete the survey within the 2 weeks immediately following the conclusion of the respective datathon, and the survey remained open for 3 weeks. Participant emails were collected to ensure that no individual filled out the survey multiple times but were removed prior to analysis. The optional, opt-in survey asked respondents questions pertaining to team demographics, medical education status, medical specialty interest, familiarity with technical and computational tools, and subjective datathon quality. The questions were divided between 4 survey pages, each taking approximately 1 minute to complete; no partial survey responses were submitted. Participants were asked to rate their familiarity with quantitative tools before and after the datathon on a 4-point scale (1=no familiarity, 4=a lot of familiarity) and were also informed that the study results would be anonymized and deidentified prior to analysis. To assess the efficacy of the aforementioned technical Python and R tutorials for datathon participants, we compared them against participant subjective familiarity with quantitative tools—namely, GitHub and Microsoft Excel—that were not taught explicitly as a part of the datathon. Data were analyzed using the Fisher exact test in Python 3. To better characterize participant experiences during the datathon, survey respondents also rated their agreement with a set of 5 standardized statements regarding (1) overall enjoyment of the datathon, (2) VBC topic understanding, (3) ability to identify problems in health care, (4) ability to generate insights from data, and (5) likelihood of future datathon participation. Participant sentiment was quantified using a 5-point Likert scale (1=strongly disagree, 5=strongly agree) [30].

### Ethical Considerations

This study was exempted by the University of Pennsylvania Institutional Review Board (protocol #856530). All opt-in participants provided informed consent prior to data collection and were not compensated for participating in our optional, opt-in survey as a part of our study. Confidentiality and privacy were maintained during data acquisition and analysis, and participants had the right to withdraw their data from the study at any time without any consequences.

## Results

### Datathon Logistics

In the 2023 datathon, 28 teams consisting of a total of 109 participants registered for the datathon, of which 13 of the initial registered teams submitted a final project, while in the 2024 datathon, 25 teams consisting of 110 participants registered for the datathon, of which 14 of the initial registered teams submitted a final project. Among the submitted projects, 7 and 8 were chosen as finalists to present at the synchronous digital showcase in 2023 and 2024, respectively. In the 2023 VBC datathon, the 7 projects addressed a variety of topics related to VBC, including chronic kidney disease underdiagnosis, the efficacy of social work referrals, and readmission rates for alcohol-related conditions, among others. Similarly, the 2024 responsible generative AI datathon featured 2 clinical documentation track teams, 3 medical education track teams, and 3 mental health track teams. We include brief descriptions of each of the finalist projects in Table 1. The final showcase was followed by the announcement of the 3 winning projects; we announced the winning teams at the end of the showcase in the 2023 datathon and 48 hours after the end of the showcase in the 2024 datathon.

**Table 1.** Sample datathon project descriptions. Descriptions of finalist datathon projects for the 2023 and 2024 MDplus datathons are shown to illustrate the diversity of project submissions from participating teams.

Theme or track	Project description
Value-based care	Minimizing chronic kidney disease (CKD) underdiagnosis using machine learning Significant association of social work referral and 30-day unplanned hospital readmission for patients with alcohol-related disorders using MIMIC^a^-IV data Can we curb frequent emergency department (ED) visits due to alcohol-related conditions? Automatic knowledge graph extraction from medical discharge notes for clinical decision support Contrast overuse in patients with renal disease: a targeted analysis Analyzing acuity as a tool for value-based care Machine learning-driven forecasting and characterization of the intensive care unit (ICU)-admitted heart failure patient population in the MIMIC-IV (version 04) database
Responsible generative AI: clinical documentation	Cost-benefit analysis of non-artificial intelligence (AI) and AI models implemented for predicting chief complaints Bridging speech documentation and clinical support through LLM^b^ automation Automating trust in AI-generated clinical notes: developing a look-up tool for real-time verification
Responsible generative AI: medical education	Using a LLM for USMLE^c^ preparation via generative AI Use of LLMs in assessing how age and gender affect model accuracy in clinical reasoning
Responsible generative AI: mental health	Reassessing specialist models: risks in fine-tuning LLMs for mental health tasks Robust text classification and grounded LLM integration for personalized mental health support Characterizing suicidal ideation subtypes in social media posts via unsupervised contrastive feature identification

a MIMIC: Medical Information Mart for Intensive Care.

b LLM: large language model.

c USMLE: United States Medical Licensing Examination

The organization-accrued cost of organizing and running the datathons was US $28 per participant, averaged over the number of participants who individually registered for the datathon regardless of whether they ultimately submitted a final project. The majority of expenses supported prize money, computing resources for participants, technical skill-based workshops, and other resources that were provided during the datathon. In our experience, most of the costs accrued were for (1) the prize money of the datathon winners and (2) honorariums for the guest judges in the finalist showcase events. We primarily relied on sponsorships from industry partners to provide computing resources for participants, and MDplus community members readily volunteered to help lead technical skill-based workshops and offer pro-bono mentorship to participating teams.

### Survey Results

Out of the 219 registered participants (summed over both datathons), 61 (28%) completed the postdatathon survey (Table 2). A majority who completed the survey identified as male (71%, 43/61) and were under the age of 25 years (61%, 37/61). Survey respondents self-reported as Asian (69%, 42/61), White (20%, 12/61), Middle Eastern or North African (3.3%, 2/61), Hispanic or Latinx (3.3%, 2/61), or Black or African American (1.6%, 1/61); and 3.3% (2/61) preferred not to say. In total, 49/61 (80%) of survey respondents were medical students (Table 3); there was a wide range of medical specialty interests amongst the medical trainee survey respondents, with internal medicine (21/49), surgery (17/49), and radiology (11/49) being the most popular specialties.

**Table 2.** Demographic information of participants who completed the postdatathon survey (N=61).

Characteristics	Value, n (%)
Age, years	
<25	37 (61)
25–30	22 (36)
30–35	1 (1.6)
≥35	1 (1.6)
Self-reported race and ethnicity	
Asian	42 (69)
Hispanic or Latinx	2 (3.3)
Middle Eastern or North African	2 (3.3)
White	12 (20)
Black or African American	1 (1.6)
Prefer not to say	2 (3.3)
Gender	
Male	43 (71)
Female	18 (29)
Sexual orientation	
Heterosexual or straight	57 (93)
Bisexual, gay, lesbian, or other	4 (6.6)
Disability status	
Does not identify as a person with a disability	54 (89)
Does identify as a person with a disability	5 (8.2)
Prefer not to answer	2 (3.3)
Current education status	
Medical student or resident physician	49 (80)
Other	12 (20)

**Table 3.** Datathon participant analysis. Current medical education status and medical specialty interest information for participants who completed the postdatathon survey (N=49) filtered by medical student and resident physician status. Note that respondents were allowed to select multiple medical specialties.

Characteristics	Value
Current medical education status, n (%)	
First-year medical student	14 (29)
Second-year medical student	19 (39)
Third-year medical student	6 (12)
Year-out medical student	4 (8.2)
Fourth-year medical student	5 (10)
Resident physician	1 (2.0)
Medical specialty interests, n (%)	
Anesthesia or critical care	9 (18)
Cardiology	1 (2.0)
Dermatology	5 (10)
Emergency medicine (EM)	4 (8.2)
Family medicine (FM)	1 (2.0)
Internal medicine	21 (43)
Mental health counseling and therapy	2 (4.1)
Neurology	9 (18)
Obstetrics and gynecology (OB/GYN)	3 (6.1)
Ophthalmology	5 (10)
Pediatrics	5 (10)
Physical medicine and rehabilitation (PM&R)	1 (2.0)
Plastic surgery	1 (2.0)
Psychiatry	8 (16)
Radiology	11 (22)
Surgery (general or unspecified)	17 (35)
Orthopedic surgery	2 (4.1)
Not currently exploring a medical specialty	1 (2.0)

**Figure 2.**
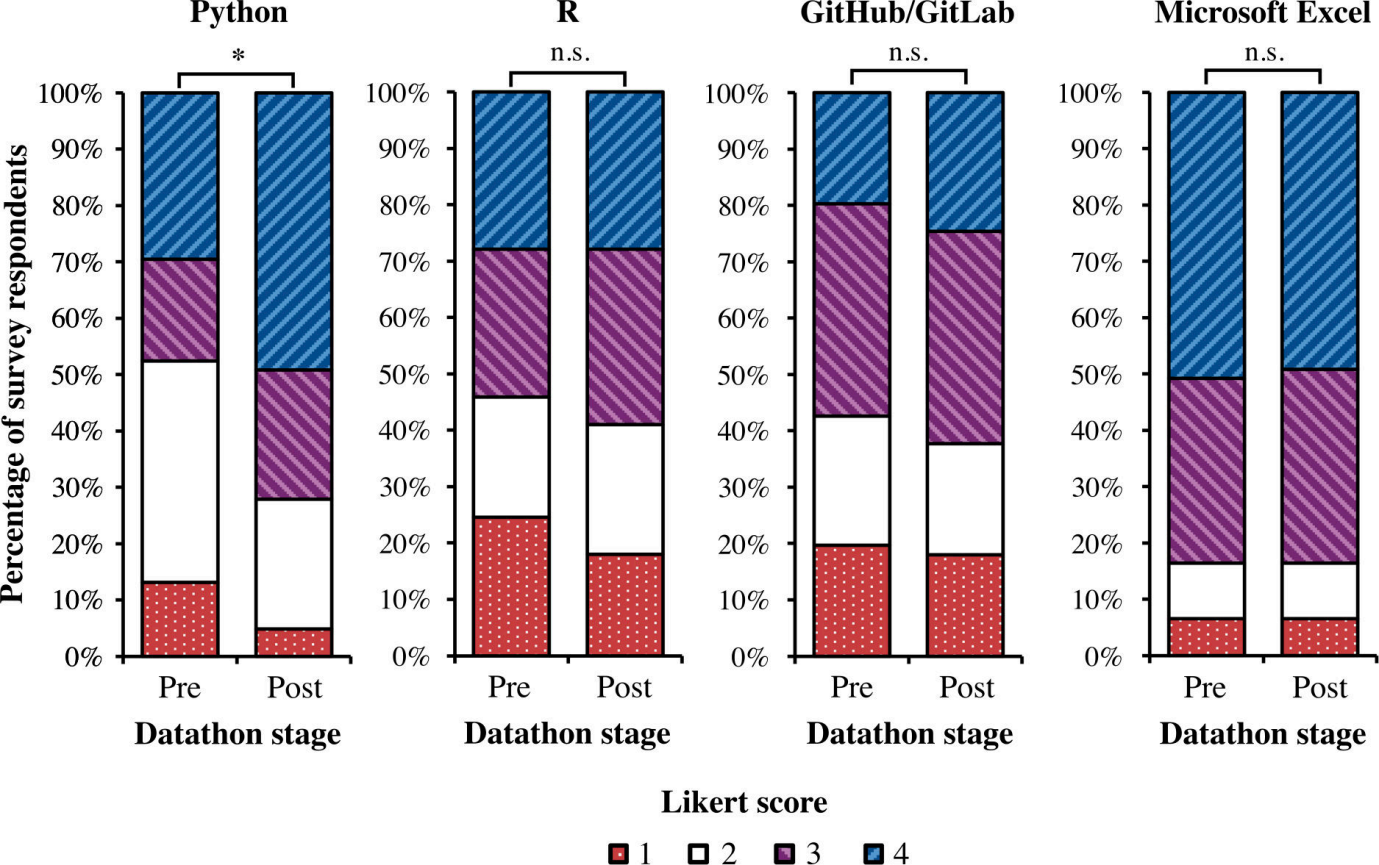
Bar plot visualizing participant self-assessment of technical skills before and after participating in the datathon for all 61 survey responses. Python was the only skill out of the 4 above that was an educational component in both the 2023 VBC and 2024 generative AI datathons. Participant scores correspond to the following: (1) no familiarity; (2) a little familiarity; (3) some familiarity; (4) a lot of familiarity. * Indicates a statistically significant difference in the distribution of scores before and after participating in the datathon (Python: *P*=.041; pairwise Fisher exact test). n.s. indicates no statistically significant difference in the distribution of scores. (R: *P*=.83; GitHub/Gitlab: *P*=.92; Microsoft Excel: *P*=1.00; pairwise Fisher exact test).

Familiarity with quantitative tools, Python, R, Github/Gitlab, and Microsoft Excel before and after participating in the datathon was assessed (Figure 2). As a reminder, a core component of the programming of both our VBC and generative AI datathons was the educational workshops and tutorials on data analysis and ML skills using Python. Workshops on the programming language R were only offered in the 2023 VBC datathon. As our negative controls, we also asked participants to rate their skills with Github/Gitlab and Microsoft Excel; neither of these software were primary educational components of the datathons. As expected, participant familiarity with GitHub/Gitlab and Microsoft Excel did not significantly change before and after the datathon (GitHub/Gitlab: *P*=.92; Microsoft Excel: *P*=1.00; pairwise Fisher exact test). In contrast, subjective participant familiarity with Python significantly improved through participation in the datathons (*P*=.04; pairwise Fisher exact test); familiarity with R showed some evidence of improvement (*P*=.83; pairwise Fisher exact test), although it did not reach the traditional threshold for statistical significance likely due to the limited sample size of the study. Our reports support that targeted educational tutorials during the datathon event can empower participants with improved technical skills relevant to data science applications in medicine.

**Figure 3.**
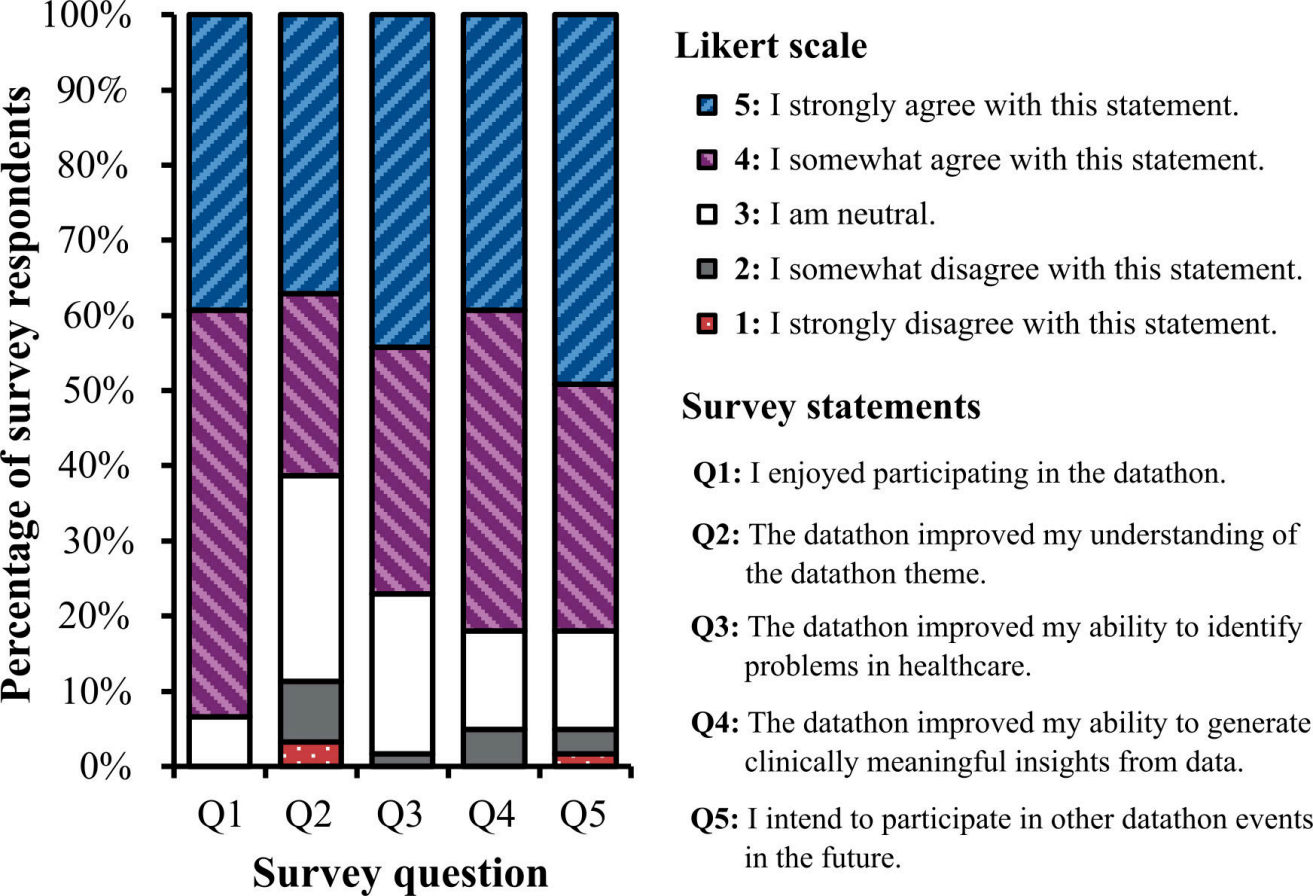
Bar plot visualizing survey results assessing for subjective datathon quality. Participant scores correspond to the following: (1) Strongly disagree; (2) Disagree; (3) Neither agree nor disagree; (4) Agree; and (5) Strongly agree.

Figure 3 examines the participant experience quantified by participant agreement with a set of standardized statements. Overall, 57/61 (93%) survey respondents enjoyed participating in the datathon, and 38/61 (62%) respondents affirmed that the datathon improved their understanding of the VBC or responsible generative AI theme (ie, Likert score of 4 or 5). We also found that 47/61 (77%) respondents stated that their ability to identify problems in health care improved, and 50/61 (82%) respondents agreed that they were better equipped to generate meaningful insights from data. Of the 61 participants, 50 participants (82%) also expressed interest in participating in similar datathon events in the future. For each of these statements, an “agreeable sentiment” was determined by indicating a Likert scale value of either 4 (“I somewhat agree with the statement”) or 5 (“I strongly agree with the statement”) on a 5-point scale in the participant survey response.

### Qualitative Survey Results

The survey also included an open-ended response option for participants to provide any additional comments. There was a mix of short, positive comments and comments that offered suggestions for future events. Based on our qualitative analysis, key areas for improvement to consider for future datathon iterations include (1) ensuring a balanced distribution of technical skills between participating teams; (2) expediting the team creation process; and (3) offering additional technical workshops and tutorials to participants. Representative example unedited participant comments are shown below:

I think in the future, it’d be more effective to make sure each team at least has a “senior” tech lead (someone with 3-5+ years of tech experience) and a “junior” tech lead (1-2 years) to ensure there is great education for all parties involved, as well as greater quality of work. This is of course for folks seeking out teams and not those who already have a team formed that they are comfortable with.

…I feel like the team creation process could’ve been a little faster and I was only able to join a team around halfway into the datathon which didn’t give us enough time to work on our idea. But overall, I really appreciate the effort and time put in by everyone involved and I definitely hope to be involved in this again!

## Discussion

### Principal Findings

In this work, we describe an instance of a trainee-led datathon to teach medical trainees how to effectively leverage modern computational tools to solve real-world problems in medicine. We show preliminary evidence that trainees become more familiar with foundational skills such as reading and writing computer programs in Python and R, are satisfied with their participation, and are eager to participate in similar initiatives in the future. To our knowledge, our national, trainee-led datathons were the first to bring together teams of medical students, residents, and graduate students to propose data-driven solutions within VBC. Our study ultimately supports that datathons can be effective platforms to teach medical trainees how to leverage AI to advance clinical medicine.

### Logistical Insights and Best Practice Recommendations

In this section, we offer additional discussion on subjective design choices and lessons learned from the MDplus datathon organizing team. We hope that our experiences and takeaways can serve as a foundation for which future datathon educational initiatives can build upon.

Perhaps one of the most notable logistical details that distinguish our datathons from related hackathons that are traditionally organized by computer science students outside of medicine is that our datathons each spanned the course of multiple weeks asynchronously, whereas hackathons are often held over the course of a few days in a single physical location. While we recognize that there are likely untapped benefits with this alternative strategy, we chose to run an extended digital datathon due to two primary reasons: (1) to support participation from MDplus members spanning multiple countries and timezones; and (2) to minimize potential time conflicts with concurrent medical school curricula for participants. In our work, these 2 constraints together necessarily precluded an in-person datathon; in situations where either one or both constraints are not limiting, future work may warrant exploring similar datathon initiatives spanning a few days hosted in a single physical location.

Separately, we also emphasize the importance of carefully choosing the datasets used in the datathon. In our 2023 VBC datathon using the MIMIC-IV dataset, we retrospectively observed that some participants initially struggled with the technical implementation details of working with the MIMIC-IV dataset due to the sheer volume of data available and the preprocessing steps before any ML modeling could be done. This consideration was especially important as the majority of participants registered in the datathon with little or no prior experience with computer programming (Figure 2). At the same time, participants also voiced enthusiasm for the diversity of data available in the MIMIC-IV dataset—making multiple modalities of data available, such as medical imaging, textual clinical documentation, biometric signals, and tabular data, allowed for participating teams to design and execute projects tailored to their specific interests. In our 2024 generative AI datathon, we found that the introduction of datathon “tracks” enabled us to offer 3 diverse dataset options while simultaneously removing the extra data processing steps outside the scope of the datathon learning objectives.

We also evaluated the utility of unstructured “office-hour” sessions where participating teams could ask experienced members of the community for assistance with their projects. Despite holding multiple office-hour sessions at different times of the day throughout the datathon, we found that only 1 team attended any of the office-hour sessions in the 2023 VBC datathon. Because of this low attendance, we opted to remove synchronous office hours from the 2024 datathon programming and instead implemented a custom anonymous discussion forum via the datathon Slack communication channel where participants could ask questions anonymously that could be viewed and answered by anyone. Subjectively, we found that this asynchronous mode of communication made it easier for participants to seek help with their projects and observed greater engagement in public discussions after this feature was implemented. Future work is warranted to more rigorously evaluate the utility of such interventions.

Finally, we acknowledge that disciplines such as medicine and computer science have historically seen disproportionate participation from trainees of certain racial, socioeconomic, and gender backgrounds. These systemic trends well described in prior work [31,32] are reproduced in our datathons as well (Table 2); as topics such as data science, VBC, and generative AI become increasingly important components of modern health care, it is crucial that all future clinicians from all backgrounds can interact meaningfully with these concepts and their applications. We hope that future work will explore how to reduce barriers to participation for historically marginalized groups of trainees.

### Related Work

The majority of prior work published in related literature details short datathons lasting a few days at a single physical location with a different target participant group. Hochheiser et al [33] describe a 2-day datathon consisting of 5 participating teams of clinicians and informaticians working on elucidating potential sources of bias within health care ML models. While their synchronous datathon model may be suitable for participants at a single physical site, such a model was intractable for our purposes as participating trainees were distributed across multiple institutions and time zones. Sobel et al [15] detail a similar datathon at a single physical location, but their study was primarily conducted with undergraduate and graduate students with pre-existing computational backgrounds, as opposed to undergraduate medical trainees from institutions granting postdoctoral fellowships as in our case. Anecdotally, we found evidence of similar initiatives held at the institutional level, such as the Digital Critical Care Datathon [34], the New York University Health Tech Datathon [35], and the Society of Critical Care Medicine Datathon [36]; each of these were single-institution initiatives with different datathon design constraints. To our knowledge, we are the first to describe a trainee-led, multi-institutional, asynchronous datathon effort and demonstrate preliminary evidence of its efficacy and potential role in the future of medical education.

### Limitations

There are also limitations associated of our study. Firstly, our datathon was coordinated digitally with participants joining from across the United States. While we acknowledge there are both benefits and drawbacks to a datathon (as opposed to their in-person counterparts), we leave a rigorous comparison between their utilities in modern medical education paradigms for future work. Furthermore, both participating in the datathon and completing the postparticipation survey were opt-in processes, and so it is unclear how our findings would translate to undergraduate medical trainees who might have systematically chosen to not participate in the datathons—for example, potential participants who were more hesitant in learning about AI and data science practices in medicine and those whose medical school coursework made concurrent participation in the datathon unfeasible. Our survey results also exclude individuals who initially signed up to express interest in participating in the datathon but ultimately decided not to submit a final project. Given the opt-in design of our survey study, we were unable to assess the efficacy of our datathons for these individuals. Future work might evaluate how similar initiatives could scale across more diverse participant profiles and foster participation from student trainees of all backgrounds and perspectives. Finally, our postparticipation survey makes use of retrospective questions that ask participants to subjectively reflect on their skill development, rather than an objective evaluation of participant skills through a standardized programming examination. We chose this study design for two primary reasons: (1) because of the diverse array of participant projects, the skill sets that they developed through their participation in the datathon are likely equally diverse, making a single standardized examination challenging to construct; and (2) in our initial efforts in organizing the datathon, we hypothesized that the survey response rate would be too low to adequately power our study if we asked participants to complete opt-in programming examinations. We leave the exploration of using more standardized assessments of programming skill competencies attained through datathon initiatives as future work.

### Conclusions

Ultimately, the goal of this datathon was to provide opportunities for trainees—especially medical students—to improve their data skills and to identify data-driven solutions to problems in health care. Participants practiced using hands-on data science and artificial intelligence to explore meaningful clinical problems and voiced a collective interest in continuing to participate in similar initiatives in the future. Overall, our results and collective experiences suggest that datathons can be valuable within undergraduate medical education.

## Supplementary material

10.2196/63602 Multimedia Appendix 1 Introduction to Python Datathon Tutorial.

10.2196/63602 Multimedia Appendix 2 Judging Rubric for Datathon Finalist Showcase Event.

10.2196/63602 Multimedia Appendix 3 Post-Datathon Survey.

## Abbreviations

LLM: large language model
MIMIC: Medical Information Mart for Intensive Care
ML: machine learning
VBC: value-based care
