# Supplementary material for: Leveraging Datathons to Teach AI in Undergraduate Medical Education: Case Study
Source: JMIR Med Educ. 2025 Apr 16;11:e63602. doi: 10.2196/63602 (PMC12017604; doi:10.2196/63602)
Supplement: Multimedia Appendix 1 [file mededu-v11-e63602-s001.pdf]

MDplus Datathon 2024

Welcome to the 2024 MDplus Datathon! We're so excited to have you participate in this year's datathon. The goal of this Python notebook is three fold:

1. **Learn** the basics of Python and how we can easily use programming as a tool to interact with and analyze medical data. **No prior Python experience is required or expected!**
2. **Understand** and interact with the clinical dataset(s) curated for this year's datathon.
3. **Write** a simple function to ask a large language model (like ChatGPT) a question using Python code.

Have any questions? Ask a question in the `#datathon-2024` MDplus Slack channel!

Python is a general-purpose, user-friendly programming language that can be used for a variety of different tasks, including analyzing clinical datasets.

While Python code is built to be easy to read and write, writing your own code to parse through datasets, multiply numbers, and other mundane tasks can be a waste of time. Instead, the Python community has already written a lot of helpful tools and functions for us to use for these tasks! These tools and functions are organized into ***packages***. We can use a Python package manager called `pip` to download some packages that are relevant for us:

```
# To run a code block like this one, press the "Play" button on the left hand
# side or press [Shift + Enter] on your keyboard while this code block is
# selected.
!pip install -U datasets ipywidgets together --quiet
```

```
472.7/472.7 kB 5.3 MB/s eta 0:00:[illegible]
139.8/139.8 kB 5.3 MB/s eta 0:00:[illegible]
68.1/68.1 kB 1.7 MB/s eta 0:00:00
116.3/116.3 kB 4.3 MB/s eta 0:00:[illegible]
134.8/134.8 kB 5.3 MB/s eta 0:00:[illegible]
2.3/2.3 MB 24.3 MB/s eta 0:00:00
194.1/194.1 kB 7.7 MB/s eta 0:00:[illegible]
1.6/1.6 MB 21.6 MB/s eta 0:00:00
```

Now that we've downloaded these packages, we also need to import them for us to use. The keyword `import` in Python tells your computer to import the packages and all of their helpful functions and tools for us to use here.

```
import datasets
import os
import requests
from google.colab import userdata, output
from ipywidgets import widgets
from together import Together
from typing import Any, Dict

output.enable_custom_widget_manager()
```

In this year's datathon, we have three separate tracks for you and your team to participate in depending on your interests.

1. Medical Education
2. Clinical Documentation
3. Mental Health

Each of these tracks have their own datasets associated with them. The following code block will download the dataset that's relevant for you depending on your track. The code itself is likely more sophisticated than what you'll need for your project, so we don't recommend worrying too much about this code block. However, if you're interested we've also commented descriptions of what the code is doing. Comments (starting with the `#` character) are just notes for humans to better understand your code, and are invisible to your computer.

More information on the datasets can be found here:
https://huggingface.co/datasets/mdplus/Datathon2024

```
# TRACK_OPTIONS is a "dictionary" that maps certain values to other values. In
# this case, our dictionary below maps the names of the Datathon tracks (e.g.,
# Medication Eduction) to the ID of the dataset in the Datathon (e.g., meded).
TRACK_OPTIONS = {
    "Medical Education": "meded",
    "Clinical Documentation": "clindoc",
    "Mental Health": "mentalhealth"
}

# TRACK is a variable that stores what track you and your team will be
# participating in.
TRACK = list(TRACK_OPTIONS.keys())[0]

# We'll create a user-friendly widget for your to be able to select your
# track that you're participating in. We create this widget using the
# Dropdown object from the `widgets` package.
track_selection = widgets.Dropdown(
    options=TRACK_OPTIONS.keys(),
    description="Track:",
    value=TRACK
)

# This is a helper function that we'll use to update the value of the
# TRACK variable depending on what you select from the dropdown widget.
# No need to look too closely at this function.
def on_change(change: Dict[str, Any]) -> None:
    global TRACK
    if change["type"] == "change" and change["name"] == "value":
        TRACK = change["new"]
    return

# Here, we're telling Dropdown widget to update the TRACK variable depending
# on the widget value.
track_selection.observe(on_change)

# Use the widget to select your Datathon track!
display(track_selection)
```

Track: Medical Education

After selecting your Datathon track from the Dropdown widget above, we can now load the dataset associated with your specific track. All of the datasets for all three tracks can be found at this link. However, manually downloading these datasets can be difficult - our datasets are quite small, but other datasets you encounter in real life can be a lot bigger!

Instead, let's use the Python `datasets` package to download our track's dataset. We'll use the `load_dataset()` function to download the dataset and save it as a variable called `ds`. We've already split your dataset into training and test splits - you should use the training split to train any models or algorithms you develop, and the test split to evaluate your project.

```
# You might get a warning about the `HF_TOKEN` not existing in your Colab
# secrets. Don't worry about this for now for the purposes of this tutorial!
ds = datasets.load_dataset("mdplus/Datathon2024", data_dir=TRACK_OPTIONS[TRACK
train_dataset = ds["train"].to_pandas()
test_dataset = ds["test"].to_pandas()
```

```
/usr/local/lib/python3.10/dist-packages/huggingface_hub/utils/_token.py:89: Us
The secret `HF_TOKEN` does not exist in your Colab secrets.
To authenticate with the Hugging Face Hub, create a token in your settings tak
You will be able to reuse this secret in all of your notebooks.
Please note that authentication is recommended but still optional to access pu
  warnings.warn(
```

What does the training dataset look like? Let's take a look!

```
train_dataset
```

	question	answer	options	meta_info	answer_idx
**0**	A 23-year-old pregnant woman at 22 weeks gesta...	Nitrofurantoin	{'A': 'Ampicillin', 'B': 'Ceftriaxone', 'C': '...	step2&3	E
**1**	A 3-month-old baby died suddenly at night whil...	Placing the infant in a supine position on a f...	{'A': 'Placing the infant in a supine position...	step2&3	A
**2**	A mother brings her 3-week-old infant to the p...	Abnormal migration of ventral pancreatic bud	{'A': 'Abnormal migration of ventral pancreati...	step1	A
**3**	A pulmonary autopsy specimen from a 58-year-ol...	Thromboembolism	{'A': 'Thromboembolism', 'B': 'Pulmonary ische...	step1	A
**4**	A 20-year-old woman presents with menorrhagia [illegible]	Von Willebrand disease	{'A': 'Factor V Leiden', 'B': 'Hemophilia A', ...	step1	E

As you can see, the dataset is a table where each row is a datapoint, and each column is a variable. To learn more about how to programmatically work with the dataset, check out this short 10-minute tutorial on the python `pandas` package here.

Now that we have our dataset downloaded, what can we do with it? One option is to feed it into a Large Language Model (LLM)! An LLM is exactly what it sounds like - a machine learning model that takes text data as input, and can process text to answer any questions or perform tasks or instructions. There are a lot of LLMs out there now, including [ChatGPT] and [Meta Llama 3.1].

Note that LLMs are also *large* - so large that it's very difficult or even possible to download these models and run on your own computer. Instead, companies like [Together AI] have dedicated supercomputers that will run any request or command that you have - all you need to do is pay a few cents per question you ask the LLM (see the pricing details [here] if you're interested). Together AI also offers $5 in free API credits when you sign up, which should be more than enough to get started.

Important: Note that for the purposes of the Datathon, you may only use open-source models with 8-billion parameters or less if you're using an LLM as a part of your project.

Let's try and run an LLM from Meta (previously Facebook) called Llama 3.1, which is offered by the Together AI platform. Follow these steps:

1. Go to [together.ai] and click "Get Started" at the upper right. Create an account.
2. Create an API key at [this link]. An API key is a unique key that tells Together AI that it's you who is making a request to their servers. **[IMPORTANT]: Never share your API key with anyone!!**
3. Copy your API key. In Google Colab, click on the "Secrets" icon on the toolbar on the left hand side of your screen (it should look like a key). Set the name of your API key to `TOGETHER_API_KEY` and paste the value of your API key to the "Value" field. Grant this Notebook access to this secret.

If all goes well, the following line of code should run without any errors.

```
# WARNING: DO NOT SHARE YOUR API KEY WITH ANYONE!
os.environ["TOGETHER_API_KEY"] = userdata.get("TOGETHER_API_KEY")
```

To "log into" Together AI and verify your identity with its platform, we need to instantiate a "client" object to establish the relevant connection details between your computer and Together AI's servers. A lot of complicated stuff happens in the background, but for us, all we need to do is run the following line:

```
llm_client = Together()
```

Normally when asking a question to a language model, we would type our question out in a search box and press [Enter], and the model's response would begin to show up on the screen. This is fine to do for general, everyday use, but what if you want to tell the language model to perform *thousands* of tasks? We can programatically ask LLMs questions using the code below.

Firstly, let's extract a sample input to the language model from your Datathon track dataset:

```
# Note that which column is selected depends on which dataset you loaded. Only
# the Medical Education dataset has a "question" column, the Clinical Documen-
# tation track a "dialogue" column, and the Mental Health track a "text" colum
if TRACK == "Medical Education":
    input_column_name = "question"
elif TRACK == "Clinical Documentation":
    input_column_name = "dialogue"
else:
    input_column_name = "text"

sample_input = train_dataset.iloc[0][input_column_name]

print(f"Input to LLM:\n{sample_input}")
```

```
Input to LLM:
A 23-year-old pregnant woman at 22 weeks gestation presents with burning upon
```

We've saved the input to the language model as a variable `sample_input`. To feed this into the LLM and ask for a response, all we need to do is run the code below. In our example, we'll use the Llama 3.1 model from Meta for this demo.

****Warning****: Running this next block of code will cost a few cents from your free $5 API credits from your Together AI account.

```
# Asking the Together AI llm_client to respond to your instruction will cost
# money! Be conscientious about any calls to llm_clients that you make.
modelID = "meta-llama/Meta-Llama-3.1-8B-Instruct-Turbo"
completion = llm_client.chat.completions.create(
    model=modelID,
    messages=[{
        "role": "user",
        "content": sample_input
    }],
)
```

That's it! We've asked the model our question, and the LLM's response is saved as the `completion` variable. We can print out the LLM's response here:

```
print(completion.choices[0].message.content)
```

```
The patient's symptoms of burning upon urination, or dysuria, are suggestive (

Given the patient's pregnancy and symptoms, the best treatment for this patie

The American College of Obstetricians and Gynecologists (ACOG) recommends trea

A more commonly used alternative in pregnancy is nitrofurantoin, which is safe

In this case, since the patient is at 22 weeks gestation, nitrofurantoin woul
```

There's a lot more things you can do with models to alter their performance and behavior - you can check out the [Together AI documentation]() and also the [API reference]() as a starting point. Prompt engineering is also an easy way to improve model performance; a good starting point is available [here]().

Frequently Asked Questions

1. **Do I need to use LLMs for my datathon project?** No, feel free to use whatever technology(s) you want.
2. **Do I need to use Python for my datathon project?** No, feel free to use whatever programming language you want.
3. **What LLMs can I use?** The full list of LLMs offered by Together AI are available [here](). Out of

these options, you may use any model in the table below. Models that are bolded are generally recommended because they were released more recently and generally perform better than non-bolded models. Using any of these models is as sample as changing out the `model` parameter value in the `llm_client.chat.completions.create()` function call above:

```
completion = llm_client.chat.completions.create(
    model="TODO: Model ID of the model you want to use",
    messages=[{
        "role": "user",
        "content": "TODO: Whatever input you want to pass into the model."
    }],
)
```

Model Name	Model ID
**Gemma Instruct (2B)**	`google/gemma-2b-it`
**Llama 3.2 3B Instruct Turbo**	`meta-llama/Llama-3.2-3B-Instruct-Turbo`
**Llama 3.1 8B Instruct Turbo**	`meta-llama/Meta-Llama-3.1-8B-Instruct-Turbo`
Llama 3 8B Instruct Turbo	`meta-llama/Meta-Llama-3-8B-Instruct-Turbo`
**Mistral (7B) Instruct v0.3**	`mistralai/Mistral-7B-Instruct-v0.3`
Mistral (7B) Instruct v0.2	`mistralai/Mistral-7B-Instruct-v0.2`
Mistral (7B) Instruct	`mistralai/Mistral-7B-Instruct-v0.1`
**Qwen 2.5 7B Instruct Turbo**	`Qwen/Qwen2.5-7B-Instruct-Turbo`
**Together StripedHyena Nous (7B)**	`togethercomputer/StripedHyena-Nous-7B`

4. **Do I have to use Together AI?** No, you are welcome to use any LLM inference provider (or run models locally!). In our experience, Together AI is easy to use and costs the least amount of money. The only requirement is that any LLM used for the datathon must be both (1) at most 8 billion parameters; and (2) open-source. This means that models like ChatGPT and Anthropic's Claude models are not permitted.
5. **How can I learn more about Python?** We've compiled some helpful resources here, and you can also check out last year's Python Tutorial here. The best way to learn more is to start writing code, and ask questions in the `#datathon-2024` Slack channel!
6. **Can I use generative AI tools and language models to help my team complete our project?** Yes, the use of generative AI tools are permitted to help you complete any parts of the project. However, you and your team are responsible for any and all outputs of generative

AI tools.

```
Start coding or generate with AI.
```
