# Supplementary material for: Leveraging Datathons to Teach AI in Undergraduate Medical Education: Case Study
Source: JMIR Med Educ. 2025 Apr 16;11:e63602. doi: 10.2196/63602 (PMC12017604; doi:10.2196/63602)
Supplement: Multimedia Appendix 3 [file mededu-v11-e63602-s003.docx]

**MD+ Datathon 2023 Survey**

**Respondent Instructions:**

The following questions are asked to enable comparisons of anonymous responses across individuals and groups in order to understand the different experiences of people with different identities. We believe it is important to ask about these intersecting identities in case individuals from certain groups are having substantively different perceptions and experiences compared to individuals from other groups.

Only MD+ executive team members will have access to the survey data. Data will be aggregated and presented so that no individuals will be identifiable from the results that are shared.

**Survey Page 1 of 4:**

1. **Age** (Required, free response)
2. **Which best describes you race/ethnicity? Please select all that apply.** (Required, select at least one)
   - American Indian or Alaska Native
   - Asian
   - Black or African American
   - Hispanic or Latinx
   - Native Hawaiian or Other Pacific Islander
   - Middle Eastern or North African
   - White
   - Prefer not to say
   - Other: (Optional, free response)
3. **Which of the following gender categories best describes how you self-identify?** (Required, select exactly one)
   - Woman
   - Man
   - Non-binary/non-conforming
   - Prefer not to respond
   - Other: (Optional, free response)
4. **What sex were you assigned at birth, such as on an original birth certificate?** (Required, select exactly one)
   - Male
   - Female
5. **Which of the following best describes you?** (Required, select exactly one)
   - Heterosexual or straight
   - Gay
   - Lesbian
   - Bisexual
   - Other: (Optional, free response)
6. **Do you identify as a person with a disability or other chronic condition?** (Required, select exactly one)
   - Yes
   - No
   - Prefer not to answer

**Survey Page 2 of 4:**

1. What is your familiarity with each of the following quantitative tools **prior to the start of the datathon**? (Required, select exactly one per row)

No familiarity A little familiarity Some familiarity A lot of familiarity

Python □ □ □ □

R □ □ □ □

GitHub/Gitlab □ □ □ □

Microsoft Excel □ □ □ □

1. What is your familiarity with each of the following quantitative tools **after participating in the datathon**? (Required, select exactly one per row)

No familiarity A little familiarity Some familiarity A lot of familiarity

Python □ □ □ □

R □ □ □ □

GitHub/Gitlab □ □ □ □

Microsoft Excel □ □ □ □

**Survey Page 3 of 4:**

This next section of questions only **pertains to current medical students and resident doctors** at the time of the start of the datathon. If you are not a current medical student or resident, please skip this section.

1. **What specialty are you currently in/considering?** Please select all that apply. (Optional, select at least zero)
   - Anesthesia/Critical Care
   - Dermatology
   - Emergency Medicine (EM)
   - Family Medicine
   - Internal Medicine
   - Medical Genetics
   - Neurology
   - Pathology
   - Psychiatry
   - Physical Medicine and Rehabilitation (PM&R)
   - Radiology (Diagnostic or Interventional)
   - Surgery
   - Obstetrics and Gynecology (OB/GYN)
   - Ophthalmology
   - Pediatrics
   - Other: (Optional, free response)
2. **What year of medical school or residency are you currently in**? (Optional, select at most one)
   - MS1
   - MS2
   - MS3
   - Year out
   - MS4
   - PGY1
   - PGY2
   - PGY3
   - PGY4
   - PGY5
   - PGY6
   - PGY7+

**Survey Page 4 of 4:**

1. **I enjoyed participating in the datathon.** (Required, select exactly one)
   - 1 – Strongly disagree
   - 2 – Disagree
   - 3 – Neutral
   - 4 – Agree
   - 5 – Strongly agree
2. **The datathon improved my understanding of value-based care (VBC).** (Required, select exactly one)
   - 1 – Strongly disagree
   - 2 – Disagree
   - 3 – Neutral
   - 4 – Agree
   - 5 – Strongly agree
3. **The datathon improved my ability to identify problems in healthcare.** (Required, select exactly one)
   - 1 – Strongly disagree
   - 2 – Disagree
   - 3 – Neutral
   - 4 – Agree
   - 5 – Strongly agree
4. **The datathon improved my ability to generate clinically meaningful insights from data.** (Required, select exactly one)
   - 1 – Strongly disagree
   - 2 – Disagree
   - 3 – Neutral
   - 4 – Agree
   - 5 – Strongly agree
5. **I intend to participate in other datathon/hackathon events in the future.** (Required, select exactly one)
   - 1 – Strongly disagree
   - 2 – Disagree
   - 3 – Neutral
   - 4 – Agree
   - 5 – Strongly agree
6. **Any other comments?** (Optional, free response)

**End of Survey**
